# Reutilization of Food Waste: One-Step Extration, Purification and Characterization of Ovalbumin from Salted Egg White by Aqueous Two-Phase Flotation

**DOI:** 10.3390/foods8080286

**Published:** 2019-07-25

**Authors:** Bin Jiang, Jiaxin Na, Lele Wang, Dongmei Li, Chunhong Liu, Zhibiao Feng

**Affiliations:** Department of Applied Chemistry, Northeast Agricultural University, NO. 600 Changjiang Road Xiangfang District, Harbin 150030, China

**Keywords:** reutilization of food waste, salted egg white, ovalbumin, extraction, aqueous two-phase flotation

## Abstract

For the purpose of reducing pollution and the reutilization of salted egg whites, which are byproducts of the manufacturing process of salted egg yolks and normally treated as waste, an aqueous two-phase flotation (ATPF) composed of polyethylene glycols (PEG 1000) and (NH_4_)_2_SO_4_ was applied to develop a simple, inexpensive and efficient process for the separation of ovalbumin (OVA) from salted egg whites. The effects of the concentration of PEG, the concentration of (NH_4_)_2_SO_4_, the flow rate and the flotation time on the flotation efficiency (*Y*) and purity (*P*) of OVA were investigated. A response surface method (RSM) experiment was carried out on the basis of a single-factor experiment. An efficient separation was achieved using ATPF containing 5 mL of 80% PEG 1000 (*w*/*w*), 28 mL of 28% (NH_4_)_2_SO_4_ (*w*/*w*), 35 mL/min of the flow rate and 30 min of the flotation time, while 2 mL of the salted egg white solution (salted eggs white (*v*): water (*v*) = 1:4) was loaded. Under the optimal conditions, *Y* and *P* of OVA could reach 82.15 ± 0.24% and 92.98 ± 0.68%, respectively. The purified OVA was characterized by sodium dodecyl sulfate polyacrylamide gel electrophoresis (SDS-PAGE), reverse phase high-performance liquid chromatography (RP-HPLC), liquid chromatography-nano electrospray ionisation mass spectrometry (Nano LC-ESI-MS/MS), ultraviolet spectrum (UV), fluorescence spectrum (FL) and fourier transform infrared spectroscopy (FT-IR). The results indicated that the purity of OVA obtained by ATPF was satisfactory and there was no obvious difference in the structure of the OVA separated by ATPF and the standard. The results of the functional properties revealed no significant differences between OVA obtained by ATPF and the standard in oil binding capacity, viscosity, emulsibility and foam capacity.

## 1. Introduction

Millions of tons of losses and waste produced in the food processing industries every year cause economic, environmental, and nutritional problems. The effective utilization of these by-products, by translating waste and byproducts into resources through shifts in technology, ensures the sustainable development of food industries and a reduction of their environmental pollution.

Salted egg whites, a by-product in the manufacturing process of salted egg yolks, are usually discarded as waste due to the difficulty in treatment. Abandoned salted egg whites contain a high content of salt and organic, which is commonly regarded as an environmental problem whose disposal is troublesome for the food industry [1,2]. Salted egg white protein is a mixture of proteins that consists mainly of ovalbumin (OVA), lysozyme, ovotransferrin, ovomucoid [3]. OVA is the major protein in salted egg whites, accounting for approximately 54% of the total protein in salted egg whites. Containing a well-balanced amino acid composition, OVA is an excellent source of essential amino acids. It consists of 385 amino acids (45 kDa) with an isoelectric point (pI) of approximately 4.5 [4,5]. Although being well studied, the function of ovalbumin in eggs is still unknown. However, some studies suggested ovalbumin to be a storage protein [6]. In view of nutrition and function, OVA is one of the highest quality food proteins. Further, OVA has been proved to have biological activities, such as antibacterial activity, antihypertensive activity and immunomodulating activity [7]. In addition to displaying several functional properties including emulsification, heat-setting and foaming, OVA also has been used as an effective drug carrier [8]. Therefore, there is considerable technical interest in its separation if costs can be controlled. The current methods for separating OVA from egg white are mainly isoelectric precipitation [9,10], membrane [11] and chromatography [12]. In recent years, a few new materials were applied to separate OVA with good selectivity. However, aniline-doped cobalt mono-substituted silicotungstic acid was reported to separate OVA with an adsorption efficiency of 92% for OVA at pH 9 [13]. Liu [14] prepared a nano-composite based on reduced graphene oxide nanosheets functionalized with polymeric ionic liquid. It was used to selectively isolate OVA from egg white with an adsorption capacity of 917.4 mg g^−1^. Chen [15] described a three-dimensional reduced graphene oxide aerogel with embedded nickel oxide nanoparticles which was prepared by a one-step self-assembly reaction, which was proved to selectively isolate ovalbumin from chicken egg white. Nevertheless, the methods above are difficult to be used in the industry due to their low yield, expensive operating costs or high equipment requirements. In addition, most previous research focused on the separation of OVA from unsalted egg white solution. Extracting OVA from salted egg whites can reprocess and utilize the waste and byproducts in the production of new commercially valuable products, as well as support an abundant and cheap source of bioactive compounds. Therefore, it is necessary to develop an economical and simple method to separate OVA from salted egg whites.

The model of the unidirectional rising bubbles mass transfer was applied to an aqueous two-phase system (ATPS), which was called aqueous two-phase flotation (ATPF). Compared with aqueous two-phase extraction (ATPE), ATPF was considered as a novel technique with low consumption of organic solvents and high concentration coefficients [16]. Thus far, ATPF has been applied in the enrichment and separation of some biomolecules, such as protein and Chinese herbal medicines. Sankaran et al. [17] established a 1-propanol/(NH_4_)_2_SO_4_ ATPF to separate lipase from fermentation broth. Jiang et al. [18] used ATPF containing PEG 1000 and citrate to extract *α*-lactalbumin from whey. Under optimal conditions, the purification factor, purity and flotation efficiency were 5.33 ± 0.05, 96.78 ± 0.79% and 87.54 ± 0.76%, respectively. The use of ATPF to extract proteins could save process time and cost, and the use of biodegradable salts as the bottom phase could avoid environmental pollution problems. Pakhale et al. [19] used ATPF consisting of PEG and phosphate to isolate bromelain. Under optimal conditions, the purification ratio reached 4.26, and the recovery rate was 91.47%. Jiang et al. [20] used ATPF to separate antioxidant peptides from whey protein hydrolysate. The recovery rate of peptides from the top phase was 11.71% and the free radical scavenging per unit concentration was 29.18%. The result was significantly better than ATPE. Bi et al. [21] established an effective and non-polluting ATPF which isolated and enriched baicalin from Astragalus membranaceus extract. The experimental results showed that under optimal conditions of ATPF, the above method was successfully applied to the separation of baicalin, the enrichment ratio was more than 27, and the separation efficiency was 90%. Chang et al. [22] established a method for separating four flavonoids using ATPF. Under optimal conditions, the flotation efficiency was 96.03%, 92.07%, 94.27% and 90.51%, respectively. ATPF proved to be a potentially effective method for the separation of biomolecules, therefore, more and more attempts on the application of ATPF were carried out.

The aim of this study was to develop an inexpensive, simple, efficient separation and pollution-free process for the separation of OVA from salted egg whites to realize the reutilization of food waste while minimizing the environmental impact. With this goal, the separation of OVA from salted egg whites by ATPF of PEG 1000/ammonium sulphate was studied for the first time.

## 2. Materials and Methods

### 2.1. Instruments

The Waters e2695 liquid chromatograph equipped with 2998 PDA detector, C_8_ and C_18_ columns (Waters, Waters Corporation, Milford, MA, USA) was applied to detect the samples. The solution pH was measured by a PHS-3C pH meter (Shanghai Instrument Electric Scientific Instrument Co., Ltd. Shanghai, China). An AL-04 electronic analytical balance (Mettler Toledo Instruments Co., Ltd., Shanghai, China) was used for weighing. The sample was dialyzed with a 13000 Da MWCO filter (Spectrum Labs, Los Angeles, CA, USA). The glass rotameter (Shenyang Beixing flow meter factory, Shenyang, China) and buoy type oxygen inhalator (Shandong Huachen high pressure container Co., Ltd. Jinan, China) were used in the preparation of ATPF.

### 2.2. Materials

Salted eggs were purchased from the local supermarket. The OVA standard sample was purchased from Sigma (Burlington, MA, USA). Trifluoroacetic acid (TFA) and acetonitrile were chromatographically pure (Dikma, Beijing, China). Further, 1000 g·mol^−1^ polyethylene glycol (PEG 1000) was purchased from Aladdin (Shanghai, China) and ammonium sulphate and other reagents were analytical reagent grade.

### 2.3. Preparation of ATPF and Purification

**Figure 1:**
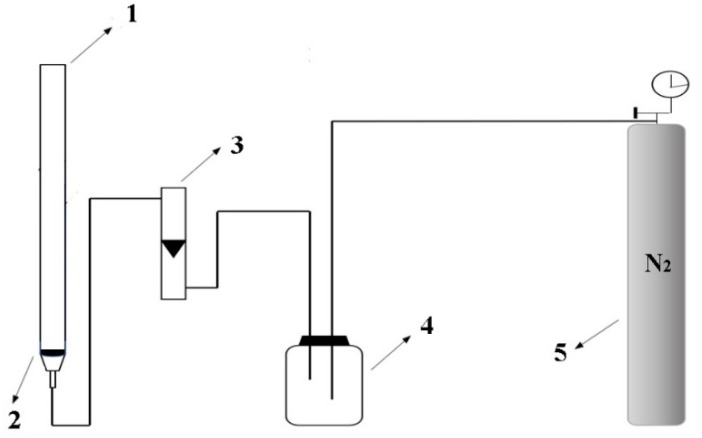
The flotation unit consisted of 1. flotation column; 2. G4 glass core; 3. flowrator; 4. gas buffer device reformed by float type oxygen inhaler; 5. high purity nitrogen cylinder.

The ATPF was prepared in a flotation column by mixing 2 mL of the salted egg white solution (salted egg white (*v*): water (*v*) = 1:4) and appropriate volume of stock solution of ammonium sulfate. The gas flow outputted from the nitrogen cylinder formed a steady flow by a gas buffer, which was adjusted by the flowrator. When the nitrogen flow rate was stable, 5 mL PEG was added. The nitrogen gas was bubbling through the bottom of the column at a certain flotation time and flow velocity. The flotation unit is described in Figure 1. The OVA was carried to the PEG phase by nitrogen gas. By measuring the *Y* and *P* in the systems, the optimum conditions, including the concentration of PEG and (NH_4_)_2_SO_4_, the flotation time and the flow rate of nitrogen, were obtained.

### 2.4. Experimental Design

The response surface method (RSM) experiment was applied to optimize the interaction between the parallel factors and separation parameters. The effects of various factors, including *X*_1_ (the mass fraction of PEG), *X*_2_ (the mass fraction of (NH_4_)_2_SO_4_), *X*_3_ (the flow rate of nitrogen) and *X*_4_ (the flotation time), were studied. The experimental design was shown in Table 1. *Y* and *P* were taken as the responses and each experiment was replicated three times

The top phase of ATPF and salted egg white solution were dialyzed against deionized water overnight by a dialysis bag with 13,000 Da molecular weight cutoffs to remove the PEG 1000 and salt. Then, the dialysate was lyophilized to obtain the purified OVA.

### 2.5. Determining of Protein

The Bradford [23] was used to determine the concentration of total protein in the top phase. RP-HPLC was used to determine the concentration of OVA in salted egg white solution and in the top phase. The waters C_8_ column (4.6 mm × 150 mm) was used and mobile phase A was 0.05% of aqueous TFA, while the mobile phase B was 0.05% TFA in acetonitrile. The flow rate was set to 1.00 mL/min while the concentration of mobile phase A decreased from 93% to 30% in 17 min and increased to 93% before 22 min [24]. The effluent was detected at a wavelength of 280 nm.

### 2.6. Definition of the Distribution of Protein in ATPF

The flotation efficiency (*Y*) and purification (*P*) of OVA were used to evaluation the enrichment and separation of OVA by ATPF, and were calculated by the following equations:

(1) $$Y=\frac{C_{\mathrm{OVA}}\times V_{\mathrm{Top}}}{C_{\mathrm{Sample}}\times V_{\mathrm{Sample}}}\times 100\%$$

(2) $$P=\frac{C_{\mathrm{OVA}}\times V_{\mathrm{Top}}}{C_{\mathrm{Top}}\times V_{\mathrm{Top}}}\times 100\%$$

where *C*_OVA_, *C*_Sample_ and *C*_Top_ were the concentrations of OVA in the top phase and salted egg white solution and total protein in top phase, respectively; while *V*_Top_ and *V*_Sample_ were the volume of the top phase and salted egg white solution, respectively.

### 2.7. Characterization of Ovalbumin Structure

#### 2.7.1. Electrophoresis

The proteins were identified by sodium dodecyl sulfate polyacrylamide gel electrophoresis (SDS–PAGE) in a gel of 12% bis-acrylamide homogeneous. The constant voltage was set at 80 V for approximately 20 min for stacking the gel and at 120 V for 55 min to separate the gel. At the end of the electrophoresis, the tape was stained with Coomassie Brilliant Blue R-250 for 30 min and decolorized with eluent [25].

#### 2.7.2. Nano LC-ESI-MS/MS

The digested protein sample was carried out by an HPLC system with a C18 column (75 μm × 80 mm). The HPLC solvent A was 9.5% water, 90% acetonitrile, and 0.5% formic acid. The HPLC solvent B was 97.5%water, 2% acetonitrile, and 0.5% formic acid. The gradient elution process was described as follows. The concentration of mobile phase A was improved from 2% to 90% within 60 min. The column flow rate was approximately 800 nanoliter per minute after splitting. The typical sample injection volume was 3 µL. The HPLC system was coupled with a linear ion trap mass spectrometer (LTQ, Thermofisher, Shaihai, China), therefore, a sample eluted from the HPLC column was directly ionized by an electrospray ionization (ESI) process and entered into the mass spectrometer. The ionization voltage was often optimized in the instrument tuning process and it ranged from 1.5 kv to 1.8 kv. The capillary temperature was set at 100 °C. The mass spectrometer was set at the data-dependent mode to acquire MS/MS data via a low energy collision induced dissociation (CID) process. The default charge state was 3 and the default collision energy was 33%. One full scan with one microscan with a mass range of 350 amu to 1650 amu was acquired, followed by nine MS/MS scans of the nine most intense ions with a full mass range and three microscans. The dynamic exclusion feature was set as follows: Repeat the count of 1 and the exclusion duration of 1 min. The exclusion width was 4Da [26].

#### 2.7.3. Spectrum Analysis

The OVA structure was analyzed using a fluorescence spectrometer PerkinElmer LS55. After being dissolved in 10 mmol/L phosphate buffer (pH = 7.2) and diluted to the appropriate concentration. The sample was measured at fluorescence excitation wavelengths of 295 nm and 280 nm, respectively. The emission spectrum was scanned from 315 nm to 415 nm. The fluorescence spectroscopy was performed at both slit and excitation slit widths of 5 nm. The ultraviolet spectrophotometer UV-2550 was used to scan the sample solution in the range of 250–360 nm.

Then, 2 mg of the sample powder was mixed with 200 mg of potassium bromide powder, which was ground under an infrared baking lamp for 10 min and pressed in a mold. The FT-IR spectra were measured at 400 cm^−1^~4000 cm^−1^. The experiment was repeated three times. After the infrared spectra was obtained, the protein conformation was analyzed by Peak Fit v4.12 software [27].

### 2.8. Determination of Functional Properties of Ovalbumin

#### 2.8.1. Oil Binding Capacity (OBC)

Further, 0.100 g of ovalbumin sample and 1 mL of edible oil were mixed in a 10 mL centrifuge tube by shaking, and the mixture was centrifuged at 716× *g* for 30 min, which was weighed as *W*_1_. The top layer of oil was removed and the residual mixture and the tube weighed as *W*_2_. The quality of the sample was recorded as *W*_Sample_. The formula for calculating was as follows:

(3) $$\mathrm{OBC}\;=\;\frac{W_{1}\;-{\;W}_{2}}{W_{\mathrm{sample}}}\;\times 100\%$$

#### 2.8.2. Viscosity

The sample solution with a concentration of 10 g/L was prepared in a 30 °C water bath, which was determined by a digital viscometer.

#### 2.8.3. Emulsibility

The measurement was carried out according to the method of Xiong [28]. The sample was dissolved in a 10 mmol/L phosphate buffer solution and the final concentration of the sample was 5 mg/mL. The protein solution was mixed with soybean oil by a volume ratio 9:1 (oil:protein solution) and placed in a high-speed homogenizer at 10,000 rpm for 1 min. Further, 50 μL of the above sample was diluted 100 times with 0.1% (*w*/*v*) SDS, and then measured by an ultraviolet-visible spectrophotometer at 500 nm. The formula for calculating the emulsifying index (*EAI*, m^2^/g) was as follows:

(4) $$EAI=\;\frac{4.303\;\times A\;\times DF}{1000\;\times C\;\times \;\varphi \;\times \;\theta }$$

where *C* was the concentration of protein, *DF* was the dilution factor (100), *θ* was the proportion of the oil phase in the emulsion (1:10), *φ* was the cuvette optical path (1 cm) and *A* was the absorbance value.

#### 2.8.4. Foam Capacity 

Furthermore, 100 mL of 1% (*w*/*v*) OVA solution was added into a 250 mL beaker, which was dispersed by a high-speed disperser at 10,000 r/min for 2 min. Then, the volume of foam was measured. The foaming capacity (*FC*) was calculated as follows [29]:

(5) $$FC=\;\frac{\left(V_{0}\;-100\right)}{100}\;\times 100\%$$

where *V*_0_ was the total volume of liquid and foam at the time of dispersion stop (mL); 100 was the volume of the stock solution (mL).

## 3. Results and Discussion

### 3.1. Single-Factor Variable Analysis

**Figure 2:**
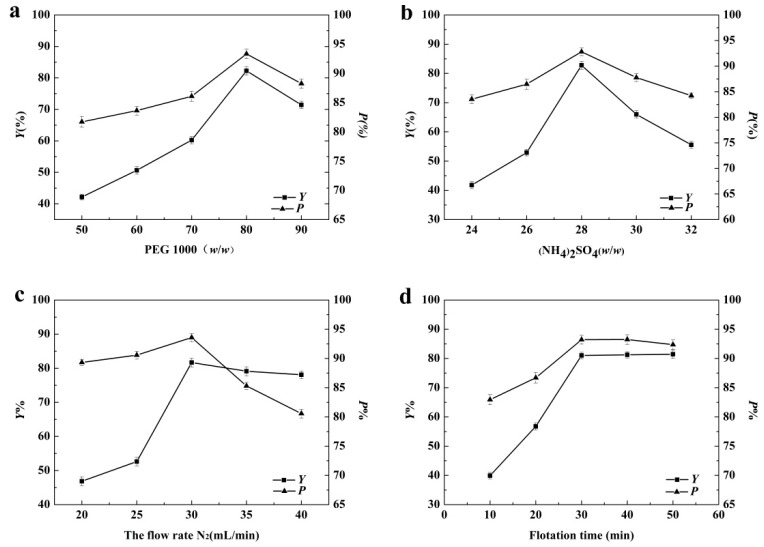
The effect of the concentration of PEG 1000 (**a**), the concentration of (NH_4_)_2_SO_4_ (**b**), the flow rate of nitrogen (**c**) and the flotation time (**d**) on the flotation.

The effect of the mass fraction of PEG 1000 and (NH_4_)_2_SO_4_, the flow rate of nitrogen and the flotation time were chosen for this study. Based on *Y* and *P*, the optimal conditions of the ATPF were chosen for further investigation. The results were shown in Figure 2.

The polymer concentration was considered to be an important factor in ATPF. The effect of the mass fraction of PEG 1000 was described in Figure 2a. The trend of *Y* and *P* changed in a similar way. When the PEG 1000 mass fraction ranged from 50% to 80%, *Y* and *P* increased. Generally, electrostatic interactions, hydrophobic interactions and the salting-out effect were the main driving forces of the protein distribution in the ATPS. The hydrophobicity of the top phase increased with the increase of the mass fraction of PEG. OVA was a hydrophobic protein that preferentially interacted with a more hydrophobic PEG-rich phase, which led to a situation that more OVA was separated into the top phase. The high viscosity was believed to be a disadvantage for the bubble entering the top phase through the interface. The viscosity of the polymer solution was affected by the polymer concentration [30], hence, the mass transfer efficiency of the biomolecule was affected. When the mass fraction of PEG was higher than 80%, the viscosity and the bubble duration in the top phase increased to an inappropriate degree and the OVA affinity to the polymer-rich top phase decreased, which caused *Y* and *P* to decrease significantly. In addition, the increasing concentration of PEG enhanced the excluded volume of the polymers, which in turn, reduced the solvent volume fraction. Thus, OVA was excluded by PEG in the top phase.

In ATPF, the ratio of the salt phase volume to the polymer phase volume was usually very large, thus high concentrations of salt was necessary for the system to sustain a stable immiscible two-phase [31]. It could be observed that the *Y* and *P* increased with the increase of the concentration of (NH_4_)_2_SO_4_ in Figure 2b. It could be attributed to the salting-out effect. The solubility of protein in the bottom phase decreased with the increase of (NH_4_)_2_SO_4_ concentration, which resulted in the transfer of protein to the top phase. When (NH_4_)_2_SO_4_ was above 28%, protein was precipitated at the phase interface, which resulted in the decrease in the *Y* and *P* [32].

The gas flow velocity significantly affects the flotation efficiency [33]. Figure 2c showed the effect of the nitrogen flow rate (20–40 mL/min) on the *Y* and *P* of OVA. Generally, the low flow rate led to the low mass transfer efficiency. The *Y* and *P* of OVA increased when the flow rate ranged from 20 mL/min to 30 mL/min, indicating that increasing of the flow rate of nitrogen made more OVA enter the top phase. At the flow rate of 30 mL/min, the maximum *Y* and *P* reached 81.66% and 93.58%, respectively. When the flow rate was above 30 mL/min, the *Y* and *P* decreased. The interface between two phases was destroyed by an excessively high flow rate and the two phases were mixed. In addition, the excessively high flow rate resulted in excessive rising speed of bubbles, causing unstable adsorption of the molecules with the bubbles and short contact time [34]. Therefore, the nitrogen flow rate of 30 mL/min was the optimum flotation condition.

The effect of flotation time on the *Y* and *P* of OVA in the range of 10–50 min was investigated and the result was described in Figure 2d. The *Y* and *P* increased with the flotation time ranging from 10 to 30 min. Before the mass transfer process was at equilibrium, the increasing flotation time facilitated full contact between the bubbles and OVA, which caused more OVA to be transferred to the top phase while the *Y* and *P* increased. When the flotation time was 30 min, the maximum *Y* and *P* were obtained, and the system basically reached the balance. There was no significant change in *Y* or *P* with the increasing of the flotation time after 30 min. Therefore, 30 min was selected as the optimum flotation condition.

From the above analysis, it could be concluded that the best single-factor conditions were as follows: PEG 80% (*w*/*w*) 5 mL, ammonium sulfate 28% (*w*/*w*) 28 mL, the flow rate of nitrogen 30 mL/min and the flotation time 30 min.

### 3.2. Response Surface Analysis

#### 3.2.1. Statistical Analysis and Model Fitting

The significant independent factors or their interactions of a multivariate complex system (e.g., ATPS) need to be analyzed by an effective mathematical statistical method [35,36]. The response surface methodology (RSM) was applied for the optimization of significant factors.

The experimental results of the response surface design were described in Table 2. The regression analysis was performed on the experimental data obtained using Design-Expert 8.0.6 software, and the equations for predicting the *Y* and *P* of OVA were obtained, which were given as follows:

*Y* = 81.81 + 4.66 × A + 1.18 × B + 4.48 × C + 8.14 × D + 1.99 × A × B + 3.86 × A × C + 3.44 × A × D + 1.39 × B × C + 2.81 × B × D − 1.42 × C × D − 14.38 × A^2^ − 13.69 × B^2^ − 14.83 × C^2^ − 8.66 × D^2^

*P* = 93.23 + 1.16 × A − 1.37 × B − 2.06 × C + 0.94 × D + 1.26 × A × B − 0.075 × A × C − 0.16 × A × D + 1.18 × B × C + 0.81 × B × D − 0.94 × C × D − 2.41 × A^2^ − 2.65 × B^2^ − 4.93 × C^2^ − 1.16 × D^2^

#### 3.2.2. Analysis of Variance

The analysis of variance (ANOVA) for the *Y* and *P* models was shown in Table 3 and Table 4, respectively. The regression models were highly significant (*p*_1_ < 0.01, *p*_2_ < 0.01), while the lack-of-fit tests were not significant (*p*_1_ = 0.0725 > 0.05, *p*_2_ = 0.3296 > 0.05). The determination coefficients (*R*_1_^2^ and *R*_2_^2^) of the predicted models were 0.9918 and 0.9558, indicating a high degree of correlation between the true and predicted values. Thus, the models explained the response adequately and could be used to analyze and predict the optimal separation conditions of OVA.

#### 3.2.3. Interactive Analysis

**Figure 3:**
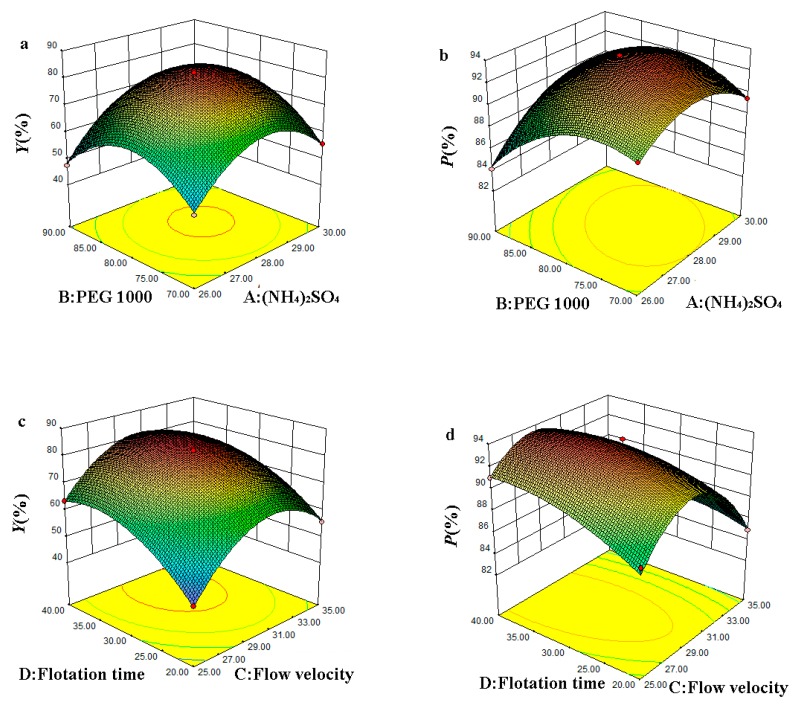
The response surface plots for *Y* (**a**,**c**) and *P* (**b**,**d**) of OVA.

The relationship between the response and experimental levels of each variable was visualized in a three-dimensional (3D) response surface plot, which provided a method to directly observe the interactions between the two test variables. The factors influencing the *Y* and *P* of OVA were shown as the response surface plots in Figure 3.

Figure 3a,b showed that *Y* and *P* first increased and then decreased with the increase of the mass fraction of (NH_4_)_2_SO_4_, when the mass fraction of PEG was at a specific level. Similarly, *Y* and *P* first increased and then decreased with the increase of the mass fraction of PEG, when the mass fraction of (NH_4_)_2_SO_4_ was at a specific level in the ATPF. The *Y* and *P* reached the maximum, when the mass fraction of PEG and (NH_4_)_2_SO_4_ reached specific levels. When the mass fraction of PEG and (NH_4_)_2_SO_4_ were set at low levels, a weak hydrophobic interaction and a weak ionic strength were obtained in the ATPF, resulting in that less OVA was separated in the top phase. The hydrophobic interaction and ionic strength gradually increased with the increase of the mass fraction of PEG and (NH_4_)_2_SO_4_, and hydrophobic OVA was separated into the top phase and other proteins were retained in the bottom phase, meaning that *Y* and *P* were increased. However, the ionic strength in the bottom phase increased and the volume of the top phase decreased with the mass fraction of PEG and the (NH_4_)_2_SO_4_ increased, causing the protein to precipitate at the interface of the two phases.

The effect of the interaction between the flow rate and the flotation time on the *Y* and *P* of OVA were shown in Figure 3c,d. It could be inferred that the higher flow rate could reduce the flotation time required to reach equilibrium. However, the interface of the two phases was destroyed when the flow rate was high. In addition, the excessively high flow rate led to excessive rising velocity of the bubbles which caused a short contact time and unstable adsorption of OVA with the bubbles. This might result in the protein adsorbed on the surface of the bubbles falling back into bottom phase. Thus, it was necessary to select the maximum flow rate and the shortest time for ATPF, under the premise of ensuring the separation effect.

#### 3.2.4. Validation of the Best Extraction Conditions

According to the results of Box-Behnken (BBD), the optimal conditions were obtained when the mass fraction of PEG 1000 and (NH_4_)_2_SO_4_, the flow rate of nitrogen and the flotation time were 79.22% (*w*/*w*), 28.50% (*w*/*w*), 29.09 mL/min and 34.59 min, respectively. Under these conditions, the *Y* and *P* value of OVA could reach 82.73% and 93.89%, respectively. In order to facilitate the operation, the predicted optimal process conditions were amended as follows: The mass fraction of PEG 1000 was 80% (*w*/*w*), the mass fraction of (NH_4_)_2_SO_4_ was 28% (*w*/*w*), the flow rate of nitrogen was 30 mL/min and the flotation time was 30 min. Under these conditions, *Y* was 82.15 ± 0.24% and *P* was 92.98 ± 0.68%. There was no significant difference between the result and the predicted value, indicating that the model was reliable.

### 3.3. Characterization of Ovalbumin Extracted Directly from Salt Egg White

**Figure 4:**
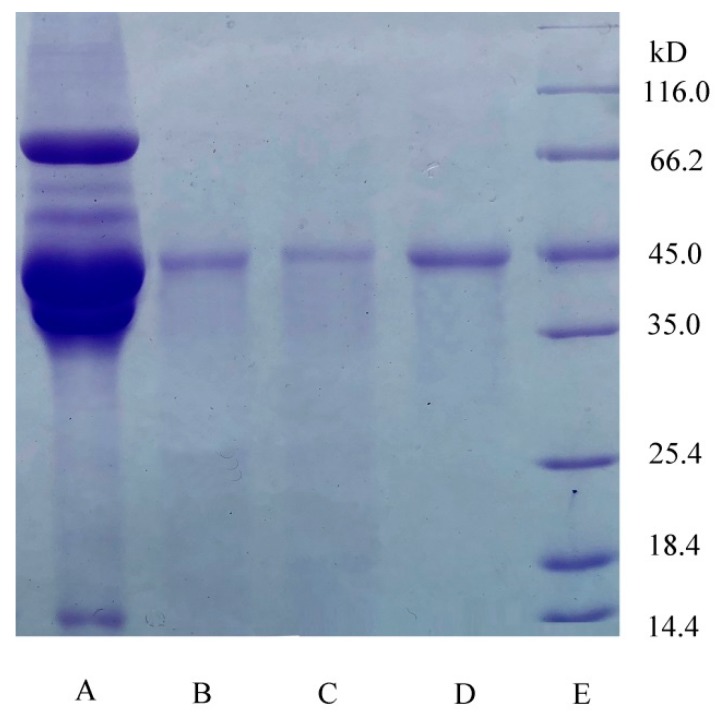
The SDS–PAGE of OVA. Lane A, the salted egg white solution (10 μg); lane B, sample (20 μg) separated by ATPF; lane C, sample (10 μg) separated by ATPF; lane D, OVA standard (10 μg); lane E, molecular mass standards.

The result of electrophoresis analysis was shown in Figure 4. The SDS–PAGE of purified OVA from the top phase of ATPF showed one band (lanes B, C) whose molecular mass was approximately 45 kDa. The bands (lanes B, C) were similar to the ovalbumin standard (lanes D). Compared with the SDS–PAGE of OVA purified from the top phase of ATPF, the salted egg white solution showed multiple bands (lane A). According to the electropherogram analysis, it was inferred that OVA could be separated to the top phase by ATPF.

**Figure 5:**
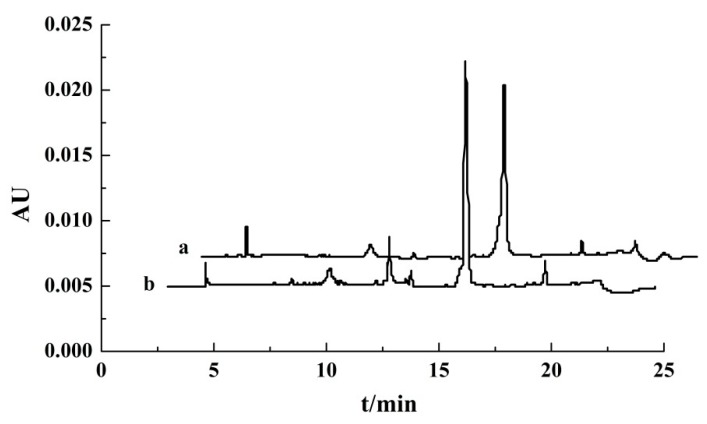
The RP-HPLC chromatograms of the OVA standard (**a**) and OVA purified from the top phase of the ATPF (**b**).

The OVA purified from the top phase of the ATPF and the OVA standard were measured by RP-HPLC, and the chromatograms were shown in Figure 5. The retention time of the main peak of the top phase in the chromatogram was approximately 13.5 min, which corresponded to the retention time of the OVA standard. Therefore, the result was consistent with electrophoresis, meaning that OVA was selectively separated to the top phase by the ATPF.

Furthermore, Nano LC-ESI-MS/MS is a reliable and sensitive method for identifying gel-separated protein tapes. Moreover, a single protein tape can be identified in mixed proteins by this method [37]. Table 5 and Table 6 showed the results of the OVA purified from the phase of ATPF determined by Nano LC-ESI-MS/MS. There were two proteins in the sample, including OVA and alpha-1-acid glycoprotein. The relative abundance of OVA was 99.4%, meaning that OVA was the main protein in the top phase of ATPF.

**Figure 6:**
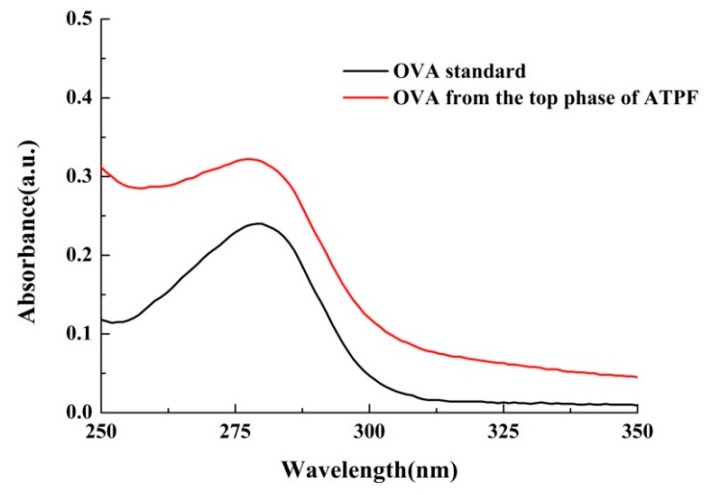
UV spectrum of samples.

**Figure 7:**
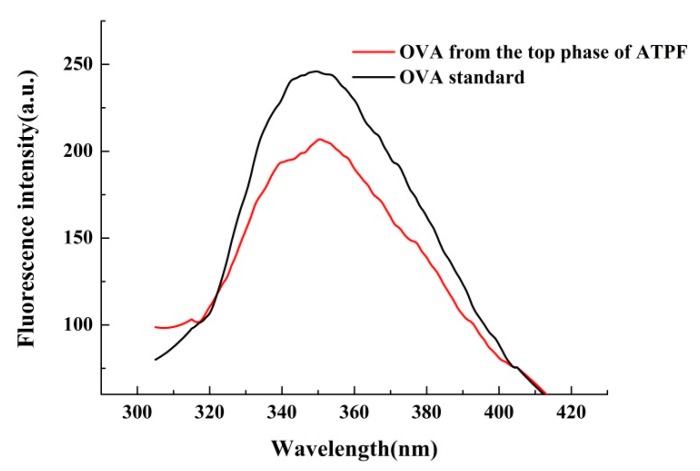
The fluorescence spectrum of the samples.

The UV spectrum of the OVA standard and OVA purified from the top phase of ATPF were shown in Figure 6. The maximum absorption peak of the ovalbumin obtained from ATPF was approximately 280 nm, which was basically consistent with the OVA standard. The same result was shown in the fluorescence analysis in Figure 7. Thus, it was inferred that there were no significant changes on the spatial structure of the ovalbumin during the separation process.

**Figure 8:**
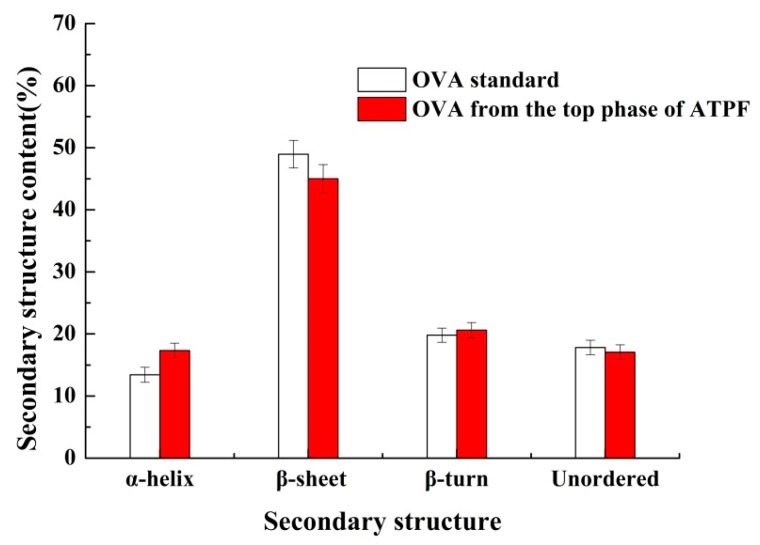
The content of the secondary structure for OVA obtained from different sources.

The secondary structure information of proteins from the top phase of ATPF was analyzed by FT-IR and PeakFit v4.12 software. The protein structure generally corresponds to the amide I absorption band between 1690 cm^−1^ and 1600 cm^−1^ in the infrared spectrum. The corresponding relationship between each sub-peak and secondary structure was as follows: Unordered (1640~1650 cm^−1^); β-turn (1660~1700cm^−1^); β-sheet (1610~1640 cm^−1^); α-helix (1650~1660 cm^−1^) [38]. Referring to the literature, PeakFit v4.12 software was used to calculate the original infrared map amide I absorption band of the OVA standard and the sample, and the fitted map was obtained after baseline correction, deconvolve Gaussian instrument response functions (IRF) deconvolution and second derivative fitting. According to the relationship between the secondary structure and sub-peak, the percentage of the secondary structure was acquired, as shown in Figure 8. It showed that unordered, β-turn, β-sheet and α-helix were not obviously changed, meaning that there was no change in the spatial structure of OVA in the ATPF separation process.

### 3.4. Determination of Ovalbumin Functional Properties

**Figure 9:**
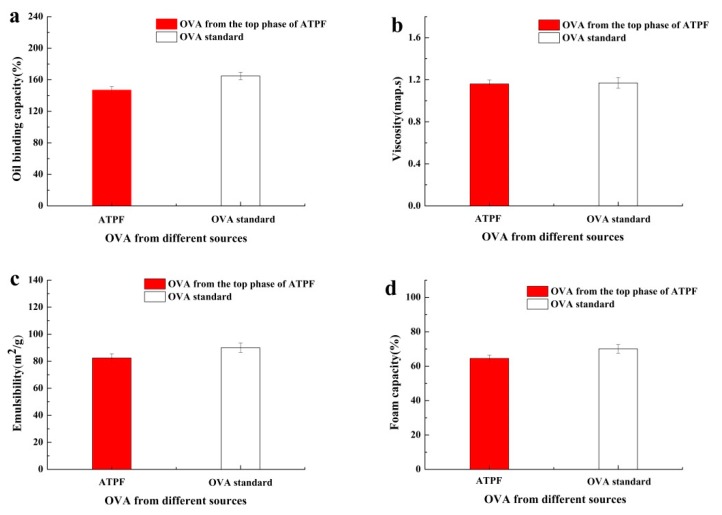
(**a**) Oil binding capacity, (**b**) viscosity, (**c**) emulsibility and (**d**) foam capacity of OVA obtained from different sources.

The results of functional properties of ovalbumin were shown in Figure 9. There were no significant differences between OVA from the top phase of ATPF and the OVA standard in an oil binding capacity, viscosity, emulsibility and foam capacity. Therefore, the spatial structure of OVA did not change excessively in ATPF and the functional properties of the protein were maintained, meaning that OVA separated from the salted egg white by ATPF could be applied in practice.

In this study, the separation and purification of OVA from salted egg whites using an ATPF consisting of PEG 1000/(NH_4_)_2_SO_4_ was investigated successfully. The optimum ATPF conditions were obtained using response surface methodology and a Box–Behnken experimental design. Accordingly, 2 mL of the salted egg white solution (salted egg white (*v*):water (*v*) = 1:4) was loaded on the ATPF. Under optimum conditions, including 5 mL of 80% PEG 1000 (*w*/*w*), 28 mL of 28%(NH_4_)_2_SO_4_ (*w*/*w*), 30 mL/min of the flow rate and 35 min of the flotation time, the maximum *Y* and the *P* reached 82.15 ± 0.24% and 92.98 ± 0.68%, respectively. The results of SDS–PAGE, RP-HPLC, Nano LC-ESI-MS/MS, ultraviolet spectrum, fluorescence spectrum, FT-IR and functional properties showed that OVA was separated and purified by ATPF, and its functional properties were maintained in ATPF, meaning that ATPF could be a valuable protocol for the separation of OVA from salted egg whites. It might be a sustainable and effective way for the utilization of salted egg whites to reduce environmental pollution.

## Figures and Tables

**Table 1:** The factors and levels in the response surface design for optimizing the separation of ovalbumin (OVA) by aqueous two-phase flotation (ATPF).

Variables	Coded Variable Levels		
	−1	0	1
*X*_1_ PEG1000 (*w*/*w)*%	70	80	90
*X*_2_(NH_4_)_2_SO_4_ (*w*/*w)*%	26	28	30
*X*_3_ flow rate of nitrogen (mL/min)	25	30	35
*X*_4_ flotation time (min)	20	30	40

**Table 2:** Box-Behnken (BBD) and the results (means of triplicate tests) for the recovery yield and purification factor of ovalbumin (OVA).

Number	A:PEG 1000 (*w*/*w*)%	B:(NH_4_)_2_SO_4_ (*w*/*w*)%	C:Flow Velocity (mL/min)	D:Flotatiom Time (min)	*Y* (%)	*P* (%)
1	1	0	0	-1	50.82	89.82
2	−1	1	0	0	47.44	84.12
3	0	0	0	0	82.56	93.03
4	0	0	0	0	81.15	93.06
5	0	−1	0	−1	53.43	90.61
6	0	0	0	0	81.97	93.08
7	−1	0	−1	0	48.11	86.53
8	0	1	0	−1	50.38	86.23
9	−1	0	1	0	49.26	83.13
10	0	1	1	0	60.27	83.56
11	1	1	0	0	61.97	88.88
12	0	1	−1	0	47.67	85.19
13	0	1	0	1	72.32	90.28
14	1	0	0	1	74.31	91.52
15	−1	−1	0	0	49.09	89.73
16	1	−1	0	0	55.64	89.45
17	−1	0	0	1	59.04	89.64
18	0	−1	1	0	55.31	83.56
19	0	0	−1	1	63.42	90.98
20	0	−1	−1	0	48.29	89.91
21	0	0	0	0	81.78	93.09
22	1	0	1	0	66.01	85.52
23	0	0	0	0	81.58	93.88
24	0	−1	0	1	64.13	91.41
25	1	0	−1	0	49.43	89.22
26	0	0	−1	−1	44.64	87.89
27	0	0	1	−1	55.67	84.93
28	0	0	1	1	68.76	84.27
29	−1	0	0	−1	49.31	87.28

**Table 3:** The variance analysis of the fitted quadratic polynomial prediction model of *Y*.

Source	Sum of Squares	df	Mean Square	F	*p*_1_
Model	4430.17	14	316.44	457.18	<0.0001
Residual	9.69	14	0.69	--	--
Lack of fit	8.61	10	0.86	3.20	0.1369
Pure error	1.08	4	0.27	--	--
CV%	--	--	1.38	--	--
*R*_1_^2^	--	--	0.9978	--	--

**Table 4:** The variance analysis of the fitted quadratic polynomial prediction model of *p*.

Source	Sum of Squares	df	Mean Square	F	*p*_2_
Model	306.56	14	21.90	101.07	<0.0001
Residual	3.03	14	0.22	--	--
Lack of fit	2.50	10	0.25	1.87	0.2856
Pure error	0.53	4	0.13	--	--
CV%	--	--	0.53	--	--
*R*_1_^2^	--	--	0.9902	--	--

**Table 5:** Nano LC-ESI-MS/MS results of the OVA purified from top phase of ATPF.

Hits	Protein Mass	No. of Peptide	Protein	UniprotKB Databases	Relative Abundance	Probability
1	43,195.66	17	OVA of chick	P01012	99.4%	99.0%
2	22,535.07	3	Alpha-1-acid glycoprotein of chick	Q8JIG5	0.6%	99.0%

**Table 6:** The peptide of the OVA purified from top phase of ATPF.

Scan No.	Peptide Mass	Peptide Sequence of Protein from Chick	Peptide Probability
6633	1772.89	ISQAVHAAHAEINEAGR	96.2%
6896	887.56	IKVYLPR	87.6%
6955	1554.71	AFKDEDTQAMPFR	96.6%
7045	1580.71	LTEWTSSNVMEER	93.2%
7037	943.53	DILNQITK	89.5%
7155	1354.65	PNDVYSFSLASR	93.9%
7195	1686.83	GGLEPINFQTAADQAR	94.9%
7241	2007.94	EVVGSAEAGVDAASVSEEFR	95.7%
7277	1246.62	ADHPFLFCIK	83.1%
7295	1344.73	HIATNAVLFFGR	95.7%
7490	1521.79	YPILPEYLQCVK	90.7%
7618	2280.17	DILNQITKPNDVYSFSLASR	91.1%
7661	1481.75	PVQMMYQIGLFR	92.3%
7735	2283.14	VTEQESKPVQMMYQIGLFR	91.0%
8705	2459.31	NVLQPSSVDSQTAMVLVNAIVFK	77.0%
10304	1857.96	ELINSWVESQTNGIIR	92.4%
9088	3032.51	VHHANENIFYCPIAIMSALAMVYLGAK	77.6%
